# t(8;21) Acute Myeloid Leukemia as a Paradigm for the Understanding of Leukemogenesis at the Level of Gene Regulation and Chromatin Programming

**DOI:** 10.3390/cells9122681

**Published:** 2020-12-13

**Authors:** Sophie Kellaway, Paulynn S. Chin, Farnaz Barneh, Constanze Bonifer, Olaf Heidenreich

**Affiliations:** 1 Institute of Cancer and Genomica Sciences, College of Medicine and Dentistry, University of Birmingham, Birmingham B152TT, UK; s.kellaway@bham.ac.uk (S.K.); p.chin@bham.ac.uk (P.S.C.); 2 Princess Máxima Centrum for Pediatric Oncology, Heidelberglaan 25, 3584CS Utrecht, The Netherlands; F.Barneh@prinsesmaximacentrum.nl

**Keywords:** acute myeloid leukemia, t(8, 21), RUNX1/ETO, epigenetic reprogramming, chromatin, gene regulatory networks, personalized medicine

## Abstract

Acute myeloid leukemia (AML) is a heterogenous disease with multiple sub-types which are defined by different somatic mutations that cause blood cell differentiation to go astray. Mutations occur in genes encoding members of the cellular machinery controlling transcription and chromatin structure, including transcription factors, chromatin modifiers, DNA-methyltransferases, but also signaling molecules that activate inducible transcription factors controlling gene expression and cell growth. Mutant cells in AML patients are unable to differentiate and adopt new identities that are shaped by the original driver mutation and by rewiring their gene regulatory networks into regulatory phenotypes with enhanced fitness. One of the best-studied AML-subtypes is the t(8;21) AML which carries a translocation fusing sequences encoding the DNA-binding domain of the hematopoietic master regulator RUNX1 to the *ETO* gene. The resulting oncoprotein, RUNX1/ETO has been studied for decades, both at the biochemical but also at the systems biology level. It functions as a dominant-negative version of RUNX1 and interferes with multiple cellular processes associated with myeloid differentiation, growth regulation and genome stability. In this review, we summarize our current knowledge of how this protein reprograms normal into malignant cells and how our current knowledge could be harnessed to treat the disease.

## 1. Overview

In this review, we will provide a comprehensive overview of the functional properties of the RUNX1/ETO fusion protein. We describe the t(8;21) AML sub-type and its clinical manifestations, how its structure and biochemistry differs from that of RUNX1 and how it serves as a paradigm for the in-depth analysis of AML subtypes.

## 2. What Is AML

Acute Myeloid Leukemia (AML) is a heterogeneous disease characterized by proliferation of neoplastic cells with impaired myeloid differentiation. Distinct classes of driver mutations alter the differentiation trajectory and epigenetic landscape differently and instead of following a normal trajectory, malignant cells adopt new identities distinct from normal cells which are maintained by specific gene regulatory networks that drive common and subtype-specific AML signalling and metabolic pathways. In adults, it is mainly a disease of the elderly and is rarely cured due to the high relapse rates. With few exceptions, for most AML subtypes the only treatment option is standard chemotherapy using regimes that have little changed in decades and which are often not an option for elderly patients. In the last years, research has therefore turned away from treating AML as a single disease entity and has undergone a shift towards understanding each subtype in fine molecular detail, in the hope to be able to interfere with leukemic growth in a specific fashion. These efforts have led to the development of highly effective specific inhibitors in other types of leukemia by targeting the oncoproteins causing APL (PML/RAR; ATRA) and CML (BCR/ABL; imatinib), but have failed to effectively target others in AML, such as the FLT3-ITD growth factor receptor whose mutation causes a particularly aggressive type of AML. Such failures have highlighted our lack of understanding of how different mutant proteins reprogram normal into malignant cells and which factors are required for their survival.

One type of AML, the t(8;21) and its oncogenic fusion protein, RUNX1/ETO have been studied for several decades at multiple levels. In this review, we will cover molecular aspects of RUNX1/ETO function and also focus on the insights recently obtained from system-wide multi-omics studies. These studies have uncovered a frightening complexity of mechanisms underlying the enhanced growth of AML cells, but also have shown ways to exploit the cancer’s weak points for precision therapy.

## 3. The Chromosomal Rearrangement t(8;21) Gives Rise to a Fusion Protein 

Chromosomal translocations are known as the major initiator of cell transformation in hematological malignancies, and recurrently involve transcription factor genes that regulate normal hematopoiesis [1,2]. A highly prevalent rearrangement found in AML occurs in the gene loci encoding members of the core-binding factor (CBF) complex, consisting of a heterodimeric transcription factor complex composed of a member of the RUNX DNA binding factor family (CBFα/ AML1/ RUNX1) and a non-DNA binding partner termed CBFβ [3]. Chromosomal rearrangements target both components of the CBF complex: the translocations t(8;21)(q22;q22) and t(16;21)(q24;q22) fuse part of *RUNX1* to members of the *ETO* (Eight Twenty-one) family, and the inversion inv(16)(p13;q220) together with the rarer t(16;16)(p13;q22) join *CBFB* to the myosin heavy chain gene *MYH11* [4,5]. CBF leukemia accounts for nearly 25% of pediatric AML cases, with t(8;21) alone being present in 15% of all cases. Its incidence decreases in older patients to 5% [6]. CBF leukemias are considered as good-prognosis AML, however, older patients are often subject to chemotherapy failure and relapse [7].

During embryogenesis, RUNX1 drives the endothelial to hematopoietic transition (EHT) to generate hematopoietic stem and progenitor cells (HSPCs). Depletion of RUNX1 at this stage is lethal in mice due to a total lack of hematopoiesis [8]. However, after the EHT and in adult hematopoiesis, expression of RUNX1 is not essential for the maintenance of self-renewal capacity of HSCs [9]. ETO (also known as RUNX1T1) is highly expressed in neurons, but its cellular functions in humans have been mainly identified as part of the RUNX1/ETO complex in AML. ETO-interactors include co-repressor complexes suggesting that this protein is a transcriptional repressor that is located in nuclear bodies [10,11]. Although highly expressed in the adult brain, insertional mutagenesis in the murine embryo leads to massive defects in gastrointestinal development, with no detected abnormality in hematopoietic system [12]. Thus, the precise function of ETO in various cellular contexts remains to be fully characterized.

**Figure 1:**
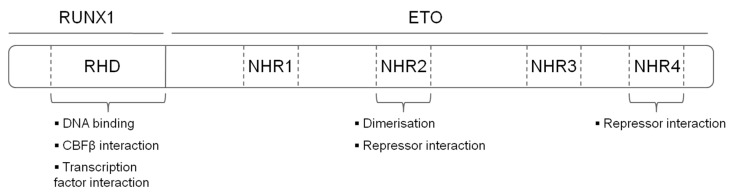
Structure and functional domains of the RUNX1/ETO fusion protein. RHD—Runt homology domain, NHR—nervy homology region.

The t(8;21) translocation fuses the N-terminal DNA binding Runt Homology Domain (RHD) domain of RUNX1 to the almost complete ETO protein creating a chimeric protein with 752 amino acids (Figure 1). The fusion protein maintains its ability to interact via its RHD with CBFβ and with DNA. ETO contributes four Nervy Homology Regions (NHR1-4) to the fusion protein. NHR1 has sequence homology to TATA-binding protein-associated factors and seems to be dispensable for gene repression by RUNX1/ETO. Nevertheless, its depletion abolishes formation of ETO nuclear bodies and suggests a role in the subcellular localization of ETO [10]. The NHR2 domain is essential for leukemogenic activity, it mediates homo and heterodimerisation with ETO members and recruits the NCoR/SIN3A corepressor together with hostone deacetylases (HDACs) [13,14,15]. Tetramerisation of the NHR2 domain itself is also essential for the leukemogenic activity of RUNX1/ETO, as mono- or dimeric fusion proteins do not efficiently bind DNA. Consequently, depletion of the NHR2 domain reverts the repressive effects of RUNX1/ETO on myeloid differentiation, and interfering with the oligomerisation by peptides abrogates the effect of RUNX1/ETO on leukemic self-renewal [16,17]. NHR4 recruits, SMRT (Silencing Mediator of Retinoic Acid and Thyroid Hormone Receptors) and SIN3, class I HDACs via nuclear receptor corepressor (NCOR) [11]. NHR3 assists NHR4 to interact with NCOR. However, binding to NCoR by NHR3 and NHR4 is not sufficient to induce maximal transcriptional repression [18]. Interestingly, a C-terminally truncated RUNX1/ETO splice variant (RUNX-ETO9a) devoid of NHR3 and NHR4 regions is highly leukemogenic when expressed at supra-physiological levels in murine, but not human HSPCs [19].

## 4. Murine Model Systems Studying t(8;21) AML—RUNX1/ETO cannot Do It Alone

Mouse models have been instrumental in elucidating the function of RUNX1/ETO in blood cell development and differentiation. One of the earliest RUNX1/ETO mouse models inserted a *RUNX1/ETO* fusion cDNA into one allele of the murine *Runx1* locus which caused an embryonic lethal phenotype. Definitive fetal liver-derived hematopoiesis was perturbed together with lethal hemorrhages indicating that also endothelial development was affected [20]. This phenotype strongly resembled that found in homozygous *Runx1* knock-out mice [21]. It was, therefore, suggested that RUNX1/ETO functions as a dominant-negative version of RUNX1. However, yolk sac cells and fetal liver cells from the heterozygous embryos were able to differentiate into macrophages and generate dysplastic multi-lineage hematopoietic progenitors in vitro [20,22]. Therefore, RUNX1/ETO plays an important driver role in leukemogenesis. To bypass the embryonic lethality of the knock-in model and to study AML development, conditional transgenic mouse models were developed that express RUNX1/ETO in adult bone marrow progenitors either using CRE/LoxP mediated recombination [23,24] or express the fusion protein under the control of the tetracycline (Tet) promoter [25]. Another inducible murine model carried RUNX1/ETO as a conditional and reversible transgene [26]. An important result from these studies was that while cells showed aberrant growth and differentiation phenotypes, RUNX1/ETO expression did not cause a full-blown AML, demonstrating that the t(8;21) mutation needs to cooperate with other mutations. This idea was confirmed when RUNX1/ETO expressing mice did succumb to AML development after exposure to the genotoxic drug N-ethyl-N-nitrosourea [24,27]. These experimental findings are in line with the detection of this translocation in Guthrie spots of newborns that were diagnosed with AML years later as adults [28]. Furthermore, the *RUNX1/ETO* transcript can be still found in the early hematopoietic stem or progenitor cells from AML patients in complete remission suggesting the existence of a RUNX1/ETO-positive pre-leukemic state [29]. Last, but not least, the majority of t(8;21) AML patients present with additional mutations that drive leukemic growth such as activating mutations in receptor tyrosine kinases which are present in at least 30% of patients with AML. When investigating the cooperating effect of expressing mutated kinases (such as mutant FLT3, RAS or KIT) together with by retrovirally transducing murine bone marrow cells with *RUNX1/ETO* into lethally irradiated syngeneic mice, all animals developed AML [30,31,32,33,34].

## 5. Molecular Aspects of RUNX1/ETO Function

The leukemic phenotype of t(8;21) AML cells is strictly dependent on the presence of RUNX1/ETO. Knockdown of the *RUNX1/ETO* transcript by siRNAs targeting the fusion site decreases the self-renewal capacity and induces differentiation [35]. A large number of studies addressed the question of how RUNX1/ETO maintains a leukemic phenotype. Histone deacetylation and gene repression via ETO mediated recruitment of co-repressors and HDACs have been suggested as the main mechanism for leukemogenic activities of RUNX1/ETO [36]. Other mechanisms for gene silencing have also been observed such as recruitment of DNMT1 (DNA Methyltransferase 1) to RUNX1 target gene promoters such as IL3 [37]. Thus, the fusion of ETO provides a docking site to introduce novel interaction partners to RUNX1 target regions and creates a shift of the balance of activation and repression [13]. Other than repressing expression or recruiting additional factors, RUNX1/ETO may also act by increasing the level of expression of other transcription factors such as FOXO1 [38] or AP-1 [39,40].

RUNX1/ETO functions as part of a stable transcription factor complex termed AML1/ETO-containing transcription factor complex (AETFC) [13]. Pull-down experiments with t(8;21) cells followed by mass spectrometry-based identification of the complex showed that RUNX1/ETO complex is enriched in RUNX1/ETO, CBFβ, FLI1/ERG, LMO2, LYL1, HDAC 1 and 2, p300, number of SRS and RBM splicing factors. Leukaemogenesis of t(8;21) is therefore not solely dependent on expression of RUNX1/ETO but its impact is the consequence of its interference with the combinatorial interplay between different hematopoietic transcriptional regulators. Interference with an expression of each of the components of this complex influences the final effect of RUNX1/ETO shifting the balance between self-renewal, proliferation and differentiation [13,41].

An interesting finding is that all t(8;21) cells always retain a wild-type allele of *RUNX1*. Knock out of the normal *RUNX1* allele in Kasumi-1 cells leads to cell cycle arrest [42] and a marked increase in sub-G1 cells and apoptosis [41]. These findings show that complete loss of RUNX1 activity is not tolerable in t(8;21) leukemia and that RUNX1 is required to counterbalance the detrimental effects of RUNX1/ETO expression.

## 6. Concurrent Mutations Add another Layer of Complexity to the RUNX1/ETO Cellular Network 

As mentioned earlier, expression of RUNX1/ETO in HSPCs blocks differentiation and enhances self-renewal capacity but leukemia develops as a result of concurrent mutations in signaling pathways and tyrosine kinases (class 1 mutations) that affect cell proliferation. The most frequent mutations occur in *KRAS, NRAS, KIT* and *FLT3* [43]. *JAK2* mutations have been also observed in therapy-induced t(8;21) AML [44]. The gain of function mutations in these genes lead to activation of PI3K/Akt, Ras/MAPK, JAK/STAT to promote transformation of *RUNX1/ETO* carrying pre-leukemic HSCs in [45]. Beside these leukemia promoting mutations in t(8;21), expression of RUNX1/ETO sensitizes the cells to acquire a hypermutable phenotype and the frequency of mutations increases when cells are exposed to sub-lethal doses of genotoxic agents or radiotherapy. Sensitization seems to be correlated with the level of RUNX1/ETO expression [27,46]. These findings urge unbiased high throughput experiments to elucidate the mutational landscape of t(8;21) for diagnostic, prognostic and therapeutic purposes [47]. Indeed, genomic profiling of adult and pediatric cases with CBF leukemia by whole genome and whole exome sequencing revealed that in t(8;21) cases, by average 11.86 ± 6.40 and 12.56 ± 6.55 mutations with functional consequences in t(8;21) were detectable in adults and pediatric patients, respectively. The rate of the mutation was higher than that of other CBF leukemia sub-type, i.e., for inv(16) which was 7.74 ± 5.13 and 7.44 ± 4.62 in adults and children. Besides known frequent mutations in *KIT, FLT3, KRAS, NRAS*, other mutations in *CCND2, MGA, TTN, KDM6A, PHIP, TET2, KMT2C, SETD2* were observed with higher frequency in t(8;21) compared to Inv(16) [48]. Notably among this list, *CCND2* (Cyclin D2) promotes proliferation of RUNX1/ETO expressing cells [49], and its mutations are frequently observed in t(8;21) AML [50]. Mutations affect the C-terminal PEST domain of CCND2 leading to increased stabilization of the protein [51]. In an international cohort of 331 t(8;21) patients with 15–84 years of age, mutations in genes regulating DNA methylation (*DNMT3, IDH2* and *TET2*) were reported to have high allele frequencies suggesting them to be among early events [47]. Other recurrent mutations were (i) those of epigenetic modifier genes including *ASXL2, EZH2*, (ii) mutations in genes encoding the cohesion complex including *SMC1A*, *SMC3* and *RAD21* which are required for cohesion of sister chromatids and DNA repair, (iii) various other defects in *CBL, DNM2, CSF3R, GIGYF2*, *ZNF687* and *ZBTB7A* [48]. Finally, mutations in the RNA helicase DHX15 affecting the spliceosome binding protein TFIP11 were also a unique feature of t(8;21) AML. Loss of function mutations in *ZBTB7A* were shown to augment the capacity for glycolysis and provide a growth advantage [52]. The spectrum of genetic mutations can also change according to clonal evolution within the same patient. By matching samples at diagnosis, remission and relapse of 19 t(8;21) patients, Christen et al. [47] observed that mutations in epigenetic and cell cycle regulators observed at diagnosis were maintained at relapse, suggesting a prominent role of these lesions in both initiation and maintenance of the disease. In contrast, mutations in RAS and tyrosine signaling pathways, the cohesion complex, or splicing associated genes were variably observed at each of the stages, demonstrating that clonal evolution of t(8;21) is dynamic during disease progression and predicts outcome.

## 7. System Wide Studies on Aberrant Chromatin Programming by RUNX1/ETO

As outlined above, the interplay of RUNX1/ETO with other factors leads to extensive reprogramming of gene regulatory networks controlling self-renewal, cell proliferation and differentiation in pre-leukemic / leukemic cells. The use of system-wide techniques such as chromatin immunoprecipitation (ChIP) assays, DNaseI hypersensitive site mapping and RNA-Sequencing is therefore essential to obtain global insights into how RUNX1/ETO blocks cell differentiation and leads to extensive changes in gene regulation. RUNX1/ETO affects the entire chromatin landscape in a dynamic fashion—not only chromatin accessibility but also hematopoietic regulator binding and histone modification patterns [36,53,54]. The mechanism behind the reorganisation of chromatin is primarily due to redistribution and displacement of RUNX1 by RUNX1/ETO [55,56,57]. These changes set up an aberrant gene regulatory network which is unique to t(8;21) leukemia, although with similarities to other core-binding factor leukemia networks, and which is underpinned by dominant AP-1 and KLF factor signatures [58]. Furthermore, other key hematopoietic regulators have notable roles in RUNX1/ETO driven leukemia—interaction with the ETS factors ERG and FLI1 influences targeting of RUNX1/ETO to chromatin as part of the AETFC complex [13,59], whilst the function of the myeloid-specific PU.1 is impaired by RUNX1/ETO, due to displacement of the AP-1 factor c-JUN [54,60]. Again, reflecting the importance of the AP-1 node in the transcription factor network, RUNX1/ETO was also recently shown to orchestrate the 3D chromatin architecture in t(8;21) cells. RUNX1/ETO participates in chromatin loops and controls the expression of other transcription factors, in this case, AP-1 and another myeloid lineage transcription factor C/EBPα, meaning its presence or absence directly leads to altered gene regulatory networks by via new enhancer–promoter interactions. RUNX1/ETO is localised predominately to open chromatin, enriched for ETS and RUNX binding sites and regulates the balance between the p300 co-activator, HDACs and acetylated histones [36,54].

The presence of repressive NHR domains in the ETO protein, together with the strong binding preference of RUNX1/ETO to active chromatin sites have historically led to RUNX1/ETO being considered as a repressor protein [61,62]. More recently, there is a growing body of evidence from global studies that RUNX1/ETO interferes with both the activating and repressive actions of RUNX1 with crucial knock-on effects in the gene regulatory networks. By inducing RUNX1/ETO in developing blood cells and assessing its impact upon the RUNX1 driven hematopoietic program a rapid (overnight) response was seen which included both activation and repression of RUNX1 target genes [56,57]. However, by inducing degradation of RUNX1/ETO in Kasumi-1 cells combined with measuring nascent RNA immediately (2 h) after depletion, the immediate early responsive targets of RUNX1/ETO could be measured. This study revealed a small core set of repressed, responsive genes that become activated once RUNX1/ETO is gone. This immediate genomic response was followed by a delayed up- and downregulation of gene expression demonstrating that the transcriptional response to RUNX1/ETO depletion seen in knock-down experiments was a mixture of direct and indirect effects due to the reprogramming of the t(8;21) transcriptional network towards differentiation [63].

The repressive roles of RUNX1/ETO are primarily related to its cooperation with co-repressors such as N-CoR and Sin3a which causes repression of myeloid genes associated with reduced histone acetylation [56,62]. Several examples of gene activity despite the presence of RUNX1/ETO on such genes also exist, such as the cell cycle genes *CCND2* and *p57^KIP2^*(also known as *CDKN1C*), and the B-cell lineage gene *PAX5* [49,64,65]. *CCND2* is de-repressed in association with AP-1 signaling, whilst *p57^KIP2^* is activated downstream of repression of KLF4—both are therefore controlled by the complex, but characteristic gene regulatory network set up by RUNX1/ETO [49,65]. It was reported that RUNX1/ETO may also be directly able to activate genes as it was found to be responsive to site-dependent p300 (also known as EP300) co-activator acetylation [66]. Whilst at many genes RUNX1/ETO blocks recruitment of p300, with twice as many mutual RUNX1/ETO/N-CoR sites as RUNX1/ETO/p300 sites [67], the RUNX1/ETO/p300 sites are not inconsequential and are associated with the expression of key genes implicated in leukaemogenesis such as KIT in cell lines [68]. However, whether there is a direct functional cooperation between RUNX1/ETO and p300 is still unclear [13]. The RUNT domain is acetylated by p300, yet complex formation appears primarily be mediated by the C-terminal portion of RUNX1 which is not present in RUNX1/ETO, although the NHR1 domain instead interacts with or is acetylated by p300 [66,69,70]. Further complicating the regulatory process is the fact that RUNX1 can interact with repressive proteins such as HDAC3 in order to repress RUNX1/ETO driven transcription [71]. Questions therefore remain regarding how RUNX1/ETO primarily represses RUNX1 transcribed myeloid genes yet the expression of signalling responsive and cell cycle associated RUNX1/ETO bound genes is maintained. The answer to this question is likely to involve the upregulation of AP-1 activity by activated signalling processes. It has indeed recently been shown that the t(8;21) specific gene regulatory network shows a strong activation of signaling responsive genes and that AP-1 activity is crucial for t(8;21) tumour development in vivo [58]. Moreover, RUNX1/ETO represses RASSF2 which is a repressor of RAS signaling leading to the chronic activation of downstream kinase activity [72]. Gene regulation by RUNX1/ETO at the chromatin level is therefore a complex process, as a consequence of mixed interactions between co-activators, co-repressors and other transcription factors.

## 8. Therapeutic Targeting of RUNX1/ETO

RUNX1/ETO-positive AML has, in general, a favourable clinical prognosis with overall survival ranging from 65% from younger adults to 75% in children but its rate of relapse is high [73]. With the exception of mylotarg, an anti-CD33 antibody–drug conjugate, targeted drugs have so far not contributed to improving the outcome. Regardless of the molecular subtype, chemotherapy has remained the cornerstone of treatment for AML for over 25 years. Induction and remission phases include the use of high dose nucleoside analogues (e.g., cytarabine), combined with anthracyclines (idarubicin or daunorubicin) [74]. Recent clinical trials may escalate the dose or duration to optimize the protocols and induce complete remission of the disease with minimal risk of relapse [75]. However, such dose escalation may not be applicable for the majority of elderly patients. Moreover, due to their longer life expectancy, children are particularly affected by long-term consequences of chemotherapy such as irreversible cardiotoxicity caused by anthracyclines urging the need for targeted therapies, urging the need for targeted therapies. The rational approaches to smart targeting of AML therefore include directly targeting the fusion protein, factors forming a complex with or being regulated by it and concurrent mutated genes that accompany the primary genetic lesion.

Leukemic fusion genes such as RUNX1/ETO constitute ideal targets for leukemia therapy. Since RUNX1/ETO initiates leukaemogenesis, all leukemic and pre-leukemic cells in a patient will harbour it. Furthermore, since it is still an essential driver of leukemia, perturbing its expression or function will affect leukemic progression. Pharmacological targeting of BCR/ABL1 by imatinib and its successors have converted CML from a deadly disease to leukemia with very good prognosis. However, targeting transcription factors has been notoriously difficult, although several recent examples such as the development of inhibitors for the formation of the RUNX1/CBFβ complex have proven the feasibility of this approach [76]. Another remarkable example is the combination therapy for acute promyelocytic leukemia with ATRA and arsenic trioxide which leads to complete and long-lasting remission in more than 90% of all patients. Interestingly, this treatment not only restores the differentiation-promoting function of the RAR moiety of the PML/RAR fusion protein, but also leads to its efficient degradation.

Current approaches to directly target RUNX1/ETO comprises the use of siRNAs targeting the fusion site of its mRNA or peptide-mediated inhibition of homo- or hetero-oligomerisation with itself and other ETO family members via its NHR3 domain [77,78,79]. Both approaches yielded very similar outcomes in cell lines with loss of self-renewal and facilitation of myeloid differentiation. Both peptides and siRNAs have poor pharmacokinetic properties complicating their therapeutic application. However, the field of therapeutic siRNA delivery is intensively investigated and the approval of two siRNA formulations for the treatment of liver-associated pathologies raises the hope that similar breakthroughs might be achievable for the therapeutic targeting of leukemic fusion transcripts.

Accompanying mutations in signal transducers such as *KIT*, *NRAS* or *KRAS* offer additional targets for therapy. However, these secondary mutations are mostly subclonal; inhibitors may therefore only affect leukemic subpopulations. Nevertheless, the safety and efficacy of dasatinib, a multi-kinase inhibitor of ABL, KIT and SRC, given sequentially after standard chemotherapy regimen and as maintenance therapy, was evaluated in CBF leukemic patients [80]. Due to the non-randomized design of the study, it was not possible to judge if the addition of dasatinib increases the efficacy of the standard regimen, However, the combination showed to be safely tolerated but the response rate among patient with *KIT* wild-type and mutated was comparable [80] encouraging an ongoing randomized phase 3 trial (NCT02013648). *FLT3* and *JAK3* mutations, although less frequent in CBF leukemia, but may result in sensitivity to inhibition by kinase inhibitors such as midastaurin, quizartinib or ruxolitinib, which, however, remains to be clinically investigated [81,82].

Other approaches with small molecules include manipulating the interaction prtners of the fusion protein. Histone deacetyases and DNA methyltransferases (DNMTs) are one of the targets that are recruited by RUNX1/ETO making HDAC and DNMT inhibitors a potential choice for targeting this sub-type of AML [83]. Studies with HDAC inhibitors showed that these drugs not only induce antileukemic effects by relieving the gene suppression by RUNX1/ETO [84], but also induce differentiation and apoptosis by the degradation of RUNX1/ETO protein [85]. DNMT inhibitors also reverse the gene expression program regulated by RUNX1/ETO, and may synergise with HDAC inhibitors [37]. Expression of RUNX1/ETO is linked with an increased mutational rate by interfering with the expression of DNA repair genes. For instance, the gene encoding 8-oxoguanine DNA Glycosylase, *OGG1*, which is a component of the base excision repair, is repressed by RUNX1/ETO [46]. This impaired DNA repair capacity has been shown to result in a particular sensitivity against Poly ADP-ribose polymerase (PARP) inhibitors [86]. Interestingly, addition of KIT inhibitors have shown to restore the sensitivity of this leukemic sub-type to PARP inhibitors [87].

Recently, we interrogated genes that are activated by RUNX1/ETO for their relevance in the RUNX1/ETO-driven leukemic programme by performing a targeted RNAi screen both in tissue culture and in vivo [49]. This screen identified *CCND2* as an essential downstream target for RUNX/1ETO. Further investigations also showed that RUNX1/ETO drives cell cycle progression in the G1 phase by also sustaining the expression of its partner protein CDK6. Notably, knockdown of *CCND2* in RUNX1/ETO-expressing AML cells yielded a gene signature that was highly similar to that of cells treated with the clinically approved CDK4/6 inhibitor palbociclib. Further evaluation both ex vivo in patient-derived AML cells and in vivo in immunodeficient mice transplanted with RUNX1/ETO-expressing AML cells demonstrated a high sensitivity of this AML subtype to palbociclib inhibition. Interestingly, similar findings have also been reported for AMLs expressing FLT3-ITD or another leukemic fusion protein NUP98/NSD1 [88,89]. Inhibition of CDK4/6 is cytostatic; ongoing research, thus, aims to identify novel drug combinations with, e.g., MTOR inhibitors or components of current chemotherapy such as cytarabine for the elimination of leukemic cells [90,91].

## 9. Outlook/Vision

RUNX1/ETO is one of the first identified and best characterized leukemic fusion proteins. The progress in understanding its functions as leukemic initiator and driver, the insights into the underlying molecular mechanisms and our understanding of the dysregulation of hematopoietic networks by this factor has provided crucial paradigms for the general understanding of leukemic fusion genes. Moreover, this increased understanding of t(8;21) AML biology is now also increasingly translated into novel, more leukemia-specific therapeutic concepts. In particular, targeting leukemic oncoproteins and components of the gene regulatory networks maintaining the leukemic phenotype is now rapidly becoming a reality [92]. However, the next logical steps of their clinical evaluation will be challenging as t(8;21) has a good response to existing chemotherapy protocols. Particularly in the paediatric setting, this creates a bottleneck for clinical testing due to the low number of refractory or relapsing patients being available for testing these new concepts. In spite of these issues, it is important to note that chemotherapy is highly genotoxic. Children and young adults will therefore be more affected by the long-term consequences of chemotherapy giving rise to secondary cancers, thus justifying clinical efforts to improve their quality of life by establishing more precise and milder approaches to treat t(8;21) AML. A successful precision medicine approach—in the true meaning of the word, not just “which toxic treatment is going to work for this patient”—would not only benefit our patients, but would serve again as a paradigm for a cure with minimal long-term burdens.
